# Corneal biomechanical changes in patients with anterior chamber inflammation: a systematic review and meta-analysis

**DOI:** 10.1007/s00417-025-06881-y

**Published:** 2025-09-11

**Authors:** Juanita Téllez-Zambrano, Germán Mejía-Salgado, Santiago Espinosa-Lugo, Paula Monje-Tobar, Alejandro Tello, Alejandra de-la-Torre

**Affiliations:** 1 Semillero de investigación Semineuros, Centro de Neurociencia NeuroVitae, Instituto de Medicina Traslacional (IMT), Escuela de Medicina y Ciencias de la Salud, Universidad del Rosario, Bogotá, Colombia; 2 Ophthalmology Interest Group-Universidad del Rosario. (OIG UR). Neuroscience (NEUROS) Research Group, Neurovitae Research Center, Institute of Translational Medicine (IMT), School of Medicine and Health Sciences, Universidad del Rosario, Bogota, Colombia; 3 Neuroscience Research Group (NEUROS), Neurovitae Center for Neuroscience, Institute of Translational Medicine (IMT), Universidad del Rosario, Bogotá, Colombia; 4 Neuroscience (NEUROS) Research Group, Neurovitae Research Center, Institute of Translational Medicine (IMT), Escuela de Medicina y Ciencias de la Salud, Universidad del Rosario, Carrera 24 # 63C-69, Bogotá, Colombia; 5 Colombian Visual Science and Translational Eye Research Insitute (CERI), Center of Excellence in Ocular Inflammation, Bogotá, Colombia; 6 Health Sciences Faculty, Universidad Autónoma de Bucaramanga UNAB, Bucaramanga, Colombia; 7 Centro Oftalmológico Virgilio Galvis, Floridablanca, Santander Colombia; 8 Universidad Industrial de Santander UIS, Bucaramanga, Colombia

**Keywords:** Corneal biomechanics, Anterior chamber inflammation, Corneal hysteresis, Corneal resistance factor, Uveitis

## Abstract

**Purpose:**

Anterior chamber inflammation (ACI) causes corneal changes like band keratopathy, reduced endothelial cell density, and increased central corneal thickness (CCT). This review analyzes ACI’s impact on corneal biomechanics.

**Methods:**

Registered in PROSPERO (CRD42024584649) following PRISMA guidelines, systematic searches were conducted in PubMed, Embase, Virtual Health Library, and MedXRiv (May 2, 2024). Studies with ≥ 10 ACI patients were included. Data on corneal hysteresis (CH), corneal resistance factor (CRF), CCT, intraocular pressure (IOP): corneal-compensated (IOPcc) and Goldmann-correlated (IOPg) were extracted. Meta-analyses assessed mean differences and heterogeneity (I²), with sensitivity analyses for outliers and moderators (age, CCT). The Mann-Whitney U Test compared age, CCT, IOPg, and IOPcc between groups.

**Results:**

Three studies comprising 126 ACI patients and 112 healthy controls were included. No significant differences were found between ACI patients and controls in age or CCT (*p* > 0.05). Meta-analyses revealed significant reductions in CH (-1.06 mmHg, 95% CI: -2.03 to -0.09, I²=83%) and CRF (-1.59 mmHg, 95% CI: -2.35 to -0.84, I²=69%) in ACI patients compared to controls. Sensitivity analysis showed that CCT as a moderator explained all the heterogeneity (R²=100%), while age did not (R²=0%). IOPg and IOPcc did not significantly differ among ACI patients (*p* = 0.051).

**Conclusion:**

Patients with ACI exhibit significant reductions in corneal biomechanical properties (CH and CRF), with CRF consistently lower across all age groups. CCT significantly impacts corneal biomechanics, potentially making biomechanics alterations more evident during remission states.

**Supplementary Information:**

The online version contains supplementary material available at 10.1007/s00417-025-06881-y.

## Key messages

*****What is known***:**

- Corneal alterations in patients with ACI include band keratopathy, reduced endothelial cell density, increased central corneal thickness, and changes in topographic parameters. Recent studies have also reported biomechanical changes, although findings remain inconsistent.

*****What is new***:**

- This study identified a significant reduction in corneal hysteresis and corneal resistance factor, indicating impaired corneal biomechanical properties in patients with ACI.
- Corneal biomechanical alterations were observed not only during active inflammation but also in inactive stages. Refractive surgeons should be aware that patients with a history of ACI may have reduced biomechanical stability, and a more detailed preoperative screening process could be necessary.

**Supplementary Information:**

The online version contains supplementary material available at 10.1007/s00417-025-06881-y.

## Introduction

Corneal biomechanics refers to the study of the mechanical properties and behavior of the cornea, such as its elasticity, stiffness, viscoelasticity, hysteresis, and resistance to deformation. These properties are critical in maintaining the cornea’s shape, directly influencing its refractive power. Abnormalities in these properties can lead to corneal weakening, increasing the risk of ectasia and irregular astigmatism [1, 2]. Various instruments have been developed to evaluate corneal biomechanics, with the Ocular Response Analyzer (ORA) from Reichert Ophthalmic Instruments in Depew, NY, being the only device that yields a metric for corneal hysteresis (CH). In addition, the ORA, a dynamic bidirectional applanation device, also measures another biomechanical property of the cornea: the corneal resistance factor (CRF) and corneal compensated intraocular pressure (IOPcc) [3, 4].

The ORA operates as a modified non-contact tonometer, measuring the corneal response to air pressure. It captures two applanation values using an infrared electro-optical system: one during corneal deformation following a rapid air pulse to a central 3 mm corneal area, and another as the cornea returns to its original shape [4, 5]. Another instrument, the CorVis ST from Oculus Optikgeräte GmbH, Wetzlar, Germany, employs corneal visualization technology. This non-contact tonometer, equipped with an ultra-high-speed Scheimpflug camera, records real-time dynamic corneal deformation after indentation with an air pulse, directly assessing corneal biomechanics [3, 4].

CH indicates the cornea’s ability to return to its original three-dimensional geometry after deformation caused by external pressure [6, 7]. CH is the difference between inward applanation pressure (during increasing air pressure) and outward applanation pressure (during decreasing air pressure). This measure represents the corneal response to dynamic deformation and is used to determine viscoelastic properties [7].

The CRF reflects the viscoelastic behavior and stiffness of the cornea and is strongly correlated with the central corneal thickness (CCT) [6, 7]. It is used to determine overall corneal resistance. Corneal biomechanics also encompasses Goldmann-correlated intraocular pressure (IOPg); IOPg is the average of inward and outward applanation pressures and is analogous to the standard contact tonometry IOP values [8, 9]. The IOPcc minimizes corneal dependence on CCT by mathematically correcting IOP values [8, 9].

Anterior chamber inflammation, as observed in conditions like anterior uveitis (AU), anterior + intermediate uveitis, and panuveitis, has been implicated in alterations of various corneal properties. These include CCT, endothelial cell parameters (such as endothelial cell density, hexagonality, and coefficient of variation) [10], and topographic parameters (such as index of surface variance, keratoconus index, root mean square spherical aberration, and posterior elevation changes [11]. Regarding biomechanics, several studies have reported reduced CH, CRF, and IOPcc in patients with ACI [12–16], suggesting corneal weakening in ACI patients.

This study aims to summarize and perform a meta-analysis of corneal biomechanics parameters in patients with ACI to provide a comprehensive understanding of the impact of inflammation in the anterior chamber on corneal biomechanics.

## Methods

A systematic review and meta-analysis were conducted following the ‘Preferred Reporting Items for Systematic Review and Meta-analysis’ PRISMA) statement (Supplementary Material 1). This review was registered in PROSPERO under the reference CRD42024584649. Institutional review board approval was not required, as this study is based on data available in the public domain and did not use individual-level data.

### Search methods for identifying studies

PubMed, Embase, Virtual Health Library, and medRxiv databases were used to conduct a systematic literature search on May 2nd, 2024. The search algorithm included a combination of terms reflecting the diseases of interest (uveitis) and (corneal biomechanics). The search strategies were modified to meet the criteria of each database using “MeSH,” “Emtree, and “DeCS” terms accordingly (Supplementary Material 2).

### Eligibility criteria for considering studies for this review

This systematic review considered primary studies involving at least 10 patients with ACI. The accepted types of studies included case series defined as Hassan Murad [17], case-control studies, cohort studies, cross-sectional studies, and clinical trials. No restriction language was applied. Excluded from consideration were non-full-text articles, studies conducted in species other than humans, and studies relying solely on secondary data sources like scoping or narrative reviews. Additionally, studies that included the target population but did not report the mean and standard deviation of biomechanical metrics required for meta-analysis (e.g., CH and CRF) were included in the qualitative synthesis but excluded from the quantitative meta-analysis.

### Criteria for participant inclusion and exclusion

In the current study, the inclusion criteria encompassed patients of all ages, genders, or ethnicities, with ACI of any etiology (infectious or non-infectious), course (acute, chronic, recurrent), and type of inflammation (granulomatous, non-granulomatous), eyes presenting any grade of ACI (cellularity or flare) as defined by The Standardization of Uveitis Nomenclature Working Group Criteria (SUN), including solely AU, intermediate uveitis with AU, and panuveitis. Patients with a previous diagnosis of glaucoma and/or cataract surgery before ACI were excluded, as it could impact biomechanical properties, generating bias.

### Primary and secondary outcomes

The primary outcome established for the study was differences in the mean of CH, CCT, CRF, IOPg, and IOPcc in patients with and without ACI. Secondary outcomes include examining the impact of demographic factors, such as age and CCT, on the corneal biomechanics of patients with ACI and exploring how the activity (active, remission) and course (acute, chronic, recurrent) of ACI impact corneal biomechanics.

### Study selection

The search results were initially downloaded in RIS format and organized using the Zotero^®^ reference manager. Subsequent steps included identifying and removing duplicate entries within Zotero^®^, complemented by verification in Microsoft Excel^®^ using authors’ names, publication titles, and DOIs. With duplicates removed, titles and abstracts retrieved were randomly assigned and independently screened by two reviewers (JTZ, PMT). The same reviewers followed a full-text review. Before screening, reviewers were trained in SUN definitions and biomechanical properties. Reviewers adhered to predefined inclusion and exclusion criteria during both screening processes, with a concordance at 98% in title and abstract screening and 100% in the full-text screening. Records were categorized as “included,” “excluded,” or “in doubt” in a Microsoft Excel^®^ database. Disagreements that arose during the paired review were resolved through further discussion among the authors. In cases where consensus remained elusive, consultation with one uveitis specialist (ADLT) was made for a final decision.

### Data collection and risk of bias assessment

The included articles were coded and downloaded using the assigned code for information extraction. The identified papers were distributed among four reviewers (JTZ, GMS, PMT, SEL), who verified each article aligned with the predefined inclusion criteria. Subsequently, the information extraction was carried out by three reviewers (JTZ, GMS, PMT) systematically in a Microsoft Excel^®^ spreadsheet, encompassing the article code, authorship details, article title, publication year, DOI, study design, the total number of patients, number of patients with ACI, number of healthy patients. Additionally, clinical characteristics of ACI—such as disease activity (active or inactive) and course (acute, chronic, or recurrent)—were also recorded.

The mean and standard deviation (SD) were collected for the following variables for both groups (ACI and healthy controls): age, CH, CRF, IOPg, IOPcc, and CCT.

The risk of bias was assessed independently by two reviewers (JTZ, PMT) using validated tools tailored to the methodological design of each article.

For cross-sectional studies, the Hoy et al. modified tool [18], which consisted of 10 items across four domains, along with a summary risk of bias assessment, was applied. The items included (1) target population, (2) sampling frame representation, (3) sample selection, (4) likelihood of nonresponse, (5) data collection source, (6) case definition, (7) parameter measurement, (8) data collection consistency, (9) follow-up period, and (10) appropriateness of numerator and denominator for the parameter of interest. The four domains are selection, nonresponse, measurement, and analysis bias. External validity was rated as “high” for scores of 0–1, “some concerns” for scores of 2, and “low” for scores of 3. Internal validity was assessed as “high” for scores of 0–2, “some concerns” for a score of 3, and “low” for a score of 4. A study was deemed to have a “high risk of bias” if either domain (internal or external validity) was rated as having a “high risk of bias.” [18].

### Data synthesis and analysis

Two authors (JTZ, SEL) performed a qualitative narrative synthesis of the relevant findings concerning changes in corneal biomechanical properties following the PRISMA guidelines [19]. Afterward, another independent author (GMS) rectified the information presented in the tables. The age and CCT of patients with ACI and healthy controls were calculated using a pooled average prevalence from studies reporting the mean and standard deviation, with statistical differences assessed using the Mann-Whitney U Test.

A mean difference meta-analysis was conducted on CH, CRF, IOPcc, and IOPg between patients with ACI and healthy controls, focusing on patient-level data rather than eye-level data, as most studies report outcomes per patient. When a study has two comparisons (the unaffected fellow eye in unilateral cases vs. healthy controls), only the variables for healthy controls were chosen for metanalysis. The mean difference approach was chosen over standardized mean difference because all included studies measured corneal biomechanics using the ORA. Only variables reported by at least two studies were included in the meta-analysis. Additionally, to assess potential confounders, variables that showed significant differences between ACI patients and healthy controls were further analyzed in separate meta-analyses, considering age and CCT differences between patients with ACI and healthy controls as moderators.

Statistical heterogeneity among the studies was assessed using the I² statistic, with thresholds defined as low (I² < 50%), moderate (I² = 50–75%), and high (I² >75%). A random effects model was chosen for all analyses, regardless of the I² value, due to the cross-sectional design of all included studies, which inherently suggested a high degree of heterogeneity. All analyses were conducted using the R package (dmetar version 0.0.9000) [20] A 95% CI was calculated for all analyses, with statistically significant results if *p* < 0.05.

In the meta-analysis, potential outliers identified through forest plot inspection were further assessed using a formal leave-one-out sensitivity analysis. Studies were excluded if their removal resulted in a substantial reduction in heterogeneity or a considerable change in the pooled effect size, indicating a disproportionate influence on both the magnitude of the effect estimate and the overall heterogeneity of the model.

### Ethical considerations

This study adheres to the ethical principles for human research established by the Helsinki Declaration, the Belmont Report, and Colombian Resolution 008430 of 1993. Due to the characteristics of our study, it does not require ethical committee approval.

## Results

A total of 372 articles were initially identified. After removing 85 duplicates, 287 articles remained. Of these, 282 were excluded after reviewing the titles and abstracts, with no additional records removed in the full-text review. Ultimately, five articles were deemed eligible for the systematic review, with meta-analysis performed on three of them.

**Fig. 1:**
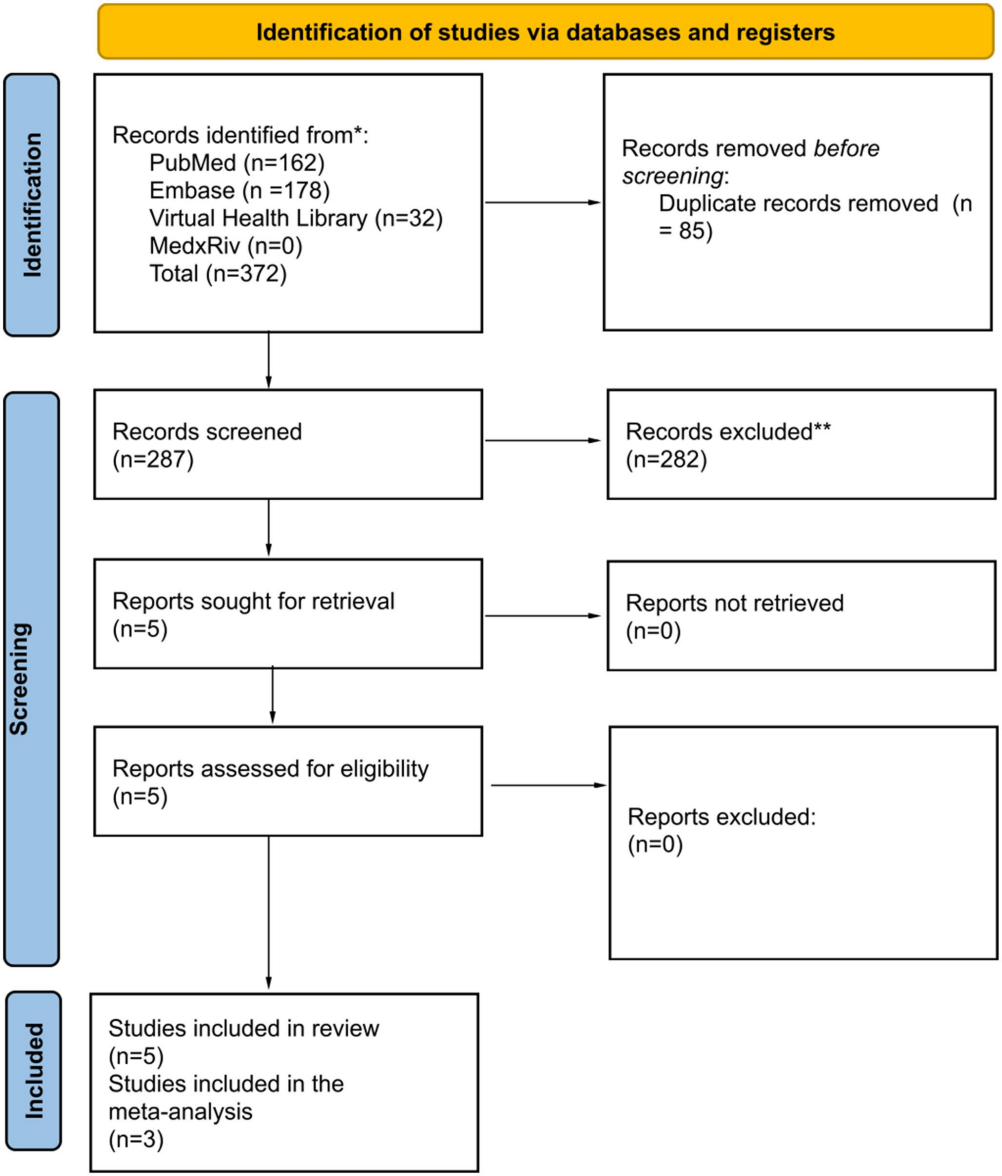
PRISMA flowchart

Among the five selected studies in the systematic review for the meta-analysis, the one published by Borrego-Sanz et al. in 2023 [14] was excluded from the meta-analysis due to the absence of data on the variables of interest, namely CH and CRF, since they did not use the ORA but another device to assess corneal biomechanics., and another study (Cankaya C et al.) was removed during a sensitivity analysis to reduce high heterogeneity (Fig. 1) [15]. Results without excluding outliers are found in Supplementary Material 3.

Among the three studies included in the meta-analysis, 126 patients with ACI and 112 healthy controls were analyzed. The ACI population had a mean age of 29.2 ± 13.0 years, with 69 (54.8%) male and 57 (45.2%) female participants; 37 of the patients were pediatric (< 18 years old). Most of the etiologies were non-infectious, including 38 cases of Fuchs uveitis syndrome (30.1%), 25 of Behçet disease (BD) (19.8%), 15 of HLA-B27 uveitis (11.9%), and nine of sarcoidosis (7.1%), among others (Table 1).

**Table 1:** Changes in corneal Biomechanical properties in patients with anterior chamber inflammation

Study, Year	Country	Population with uveitis	Control population	CH	CRF	IOPcc	IOPg
Emine Sen, 2018 * [12]	Turkey	37 patients with pediatric non-infectious uveitis **Sex**: 14 F 23 M **Mean age**: 14.2 ± 5.6 **Course**: Not specified **Activity**: Not specified **Etiology**: Pars planitis: 9/37 (24.3%) JIA: 7/37 (18.9%) BD: 6/37 (16.2%) FUS: 6/37 (16.2%) Idiopathic AU: 5/37 (13.5%) HLA-B27 AU: 3/37 (8.1%) Panuveitis: 1/37 (2.7%)	36 healthy controls **Sex**: 15 F 21 M **Mean age**: 14.4 ± 4.5	There was no difference in CH between uveitic and healthy children (10.1 ± 10.8 mmHg) *p* = 0.115	Patients with uveitis had significantly lower CRF (9.7 vs. 10.8 mmHg) *p* = 0.009	There was no difference in IOPcc between the pediatric uveitic eyes and healthy controls (13.8 ± 13.6 mmHg) *p* = 0.772	There was no difference in IOPg between the pediatric uveitic eyes and healthy controls (12.9 ± 13.9 mmHg) *p* = 0.772
Ece Turan-Vural, 2012* [13]	Turkey	51 patients with recurrent anterior uveitis. **Sex**: 26 F 25 M **Mean age**: 38.1 ± 14.5 **Course**: Recurrent 51/51 (100%) **Activity**: Inactive 51/51 (100%) **Etiology**: BD: 19/51 (37.3%) HLA-B27 AU: 12/51 (23.5%) Sarcoidosis: 9/51 (17.6%) MS: 6/51 (11.8%) Idiopathic AU: 5/51 (9.8%)	34 healthy controls **Sex**: 18 F 16 M **Mean age**: 37.7 ± 11.3	The uveitis group had significantly lower CH compared to the healthy group (8.57 ± 10.91 mmHg) *p* = 0.001	CRF was significantly lower in patients with uveitis (9.24 vs. 11.56 mmHg) *p* = 0.001	No difference was found between the two groups concerning IOPcc (15.49 vs. 15.59 mmHg) *p* = 0.798	No difference was found between the two groups concerning IOPg (13.58 vs. 13.13 mmHg) *p* = 0.194
Emine Sen, 2016* [16]	Turkey	38 patients with FUS **Sex**: 17 F 21 M **Mean age**: 35.2 ± 12.9 **Course**: Chronic 38/38 (100%) **Activity**: Not specified **Etiology**: FUS 38/38 (100%)	42 Healthy controls and 38 fellow non-affected eyes. **Sex**: 25 F 17 M **Mean age**: 32.0 ± 9.6	Compared to healthy controls, the uveitis group had lower CH (9.5 vs. 10.5 mmHg), *p* = 0.005. Compared to the fellow non-affected eyes, the uveitis group had lower CH (9.5 vs. 10.1 mmHg), *p* = 0.005	Compared to healthy controls, the uveitis group had lower CRF (9.0 vs. 10.3 mmHg), *p* = 0.001 Compared to the fellow non-affected eyes, the uveitis group, the uveitis group had lower CRF (9.0 vs. 9.9 mmHg), *p* < 0.001	Compared to healthy controls, there was no significant difference regarding IOPcc (14.8 vs. 15.0 mmHg), *p* = 0.0766 Compared to the fellow non-affected eyes, there was no significant difference regarding IOPcc (14.8 vs. 15.5 mmHg), *p* = 0.231	Compared to healthy controls, the uveitis group had lower IOPg (13.1 vs. 14.8 mmHg), *p* = 0.025 Compared to the fellow non-affected eyes, the uveitis group had lower IOPg (13.1 vs. 14.6 mmHg), *p* = 0.022
Lara Borrego-Sanz, 2023[14]	Spain	77 patients with non-infectious uveitis. **Sex**: 51 F 26 M **Mean age**: 52.68 ± 17.91 **Course**: not specified **Activity**: Active: 17/77 (22.1%) Inactive: 60/77 (77.9%) **Etiology**: HLA-B27: 14/77 (18.2%) Idiopathic AU: 13/77 (16.9%) BD: 7/77 (9.1%) MFC: 5/77 (6.5%) JIA:4/77 (5.2%) VKH: 4/77 (5.2%) Sarcoidosis: 3/77 (5.2%) Scleritis + AU:4/77 (5.2%) Pars planitis: 4/77 (5.2%) FUS: 7/77 (3.9%) Others: 15/77 (19.5%)	47 Healthy controls **Sex**: 28 F 19 M **Mean age**: 48.65 ± 13.52	Unmeasured factor	Unmeasured factor	IOPcc values in the uveitis group were statistically lower than in the control group (13.57 vs. 15.28 mmHg), *p* < 0.002	Unmeasured factor
Cem Cankaya, 2014 [15]	Turkey	34 patients with ocular BD **Sex**: 13 F 21 M **Mean age**: 33.81 ± 9.36 **Course**: not specified **Activity**: Active: 16/34 (47.1%) Inactive:18/34 (52.9%) **Course**: Not specified **Etiology**: BD: 34/34 (100%)	20 healthy controls and 18 patients with inactive BD **Sex**: 10 F 10 M Mean age: 31.05 ± 5.85	Compared to healthy controls and the inactive group, there was no significant difference regarding CH (8.51(active) vs. 8.46 (inactive) and 8.47 (healthy) mmHg), *p* > 0.05	Compared to healthy controls and the inactive group, an increase in CRF was noted in active BD (9.72(active) vs. 8.45 (inactive) vs. 8.43 (healthy), *p* < 0.05	There was no statistically significant difference in IOPcc among the groups (16.13 vs. 15.35 vs. 15.42 mmHg) *p* > 0.05	The IOPg value of the patients with active ocular BD (19.8 mmHg) was statistically higher than that of patients with inactive BD (15.89 mmHg) and the control group (15.59 mmHg), *p* < 0.05

*Studies included in the meta-analysis

*M* Male, *F* Female, *BD* Behçet’s Disease, *FUS* Fuchs Uveitis Syndrome, *MFC* Multifocal Choroiditis and Panuveitis, *JIA* Juvenile Idiopathic Arthritis, *VKH* Vogt-Koyanagi-Harada Syndrome, *CH* Corneal Hysteresis, *CRF* Corneal Resistance Factor, *IOPcc* Corneal Compensated Intraocular Pressure, *IOPg* Goldmann-Correlated Intraocular Pressure, *HLA* Human Leukocyte Antigen

There was no significant difference in age (29.2 ± 13.0 years vs. 28 ± 12.1 years; *p* = 0.827) and CCT (551 ± 3.01 μm vs. 551 ± 7.45 μm; *p* = 0.829) between ACI patients and healthy controls. Additionally, a borderline difference was found between IOPcc (14.7 ± 0.85 mmHg) and IOPg (13.2 ± 0.36 mmHg) within the ACI group (*p* = 0.051), whereas no such difference was observed in the control group (IOPg: 14.7 ± 1.02 mmHg; IOPcc: 13.0 ± 0.83 mmHg; *p* = 0.275). These findings suggest that, aside from the presence of anterior chamber inflammation, both groups were comparable in baseline characteristics.

### Study characteristics

**Fig. 2:**
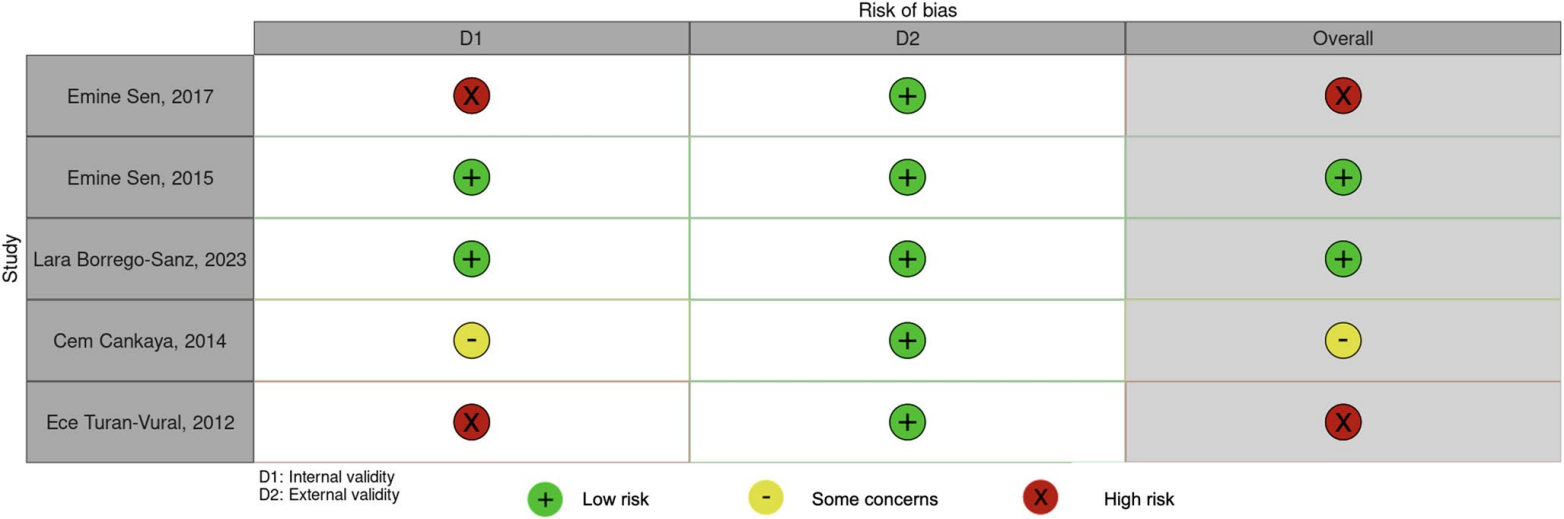
Risk of bias

Among the five included studies, all were cross-sectional and generally demonstrated a “low risk of bias,” particularly in areas such as the study population, data collection methods, case definition, measurement of parameters, consistency in data collection, and the follow-up period. However, a “high risk of bias” was identified in the likelihood of nonresponse across many of these studies. As a result, two studies were classified as having a “low risk of bias,” one study was identified with “some concerns,” and two studies were considered to have a “high risk of bias” (Fig. 2). Four studies originated from Turkey and one from Spain (Table 1).

### Qualitative synthesis on corneal biomechanics in anterior chamber inflammation

Five studies evaluating corneal biomechanical parameters in patients with ACI were included in the systematic review. Their populations, uveitis characteristics, and primary findings are summarized below.

Sen et al. (2018) [12] evaluated 37 pediatric patients (mean age: 14.2 ± 5.6 years) with non-infectious uveitis. Etiologies included pars planitis (24.3%), juvenile idiopathic arthritis (18.9%), BD (16.2%), Fuchs uveitis syndrome (16.2%), idiopathic anterior uveitis (13.5%), and HLA-B27 anterior uveitis (8.1%). Compared to 36 healthy controls, no significant differences were found in CH (10.1 ± 10.8 mmHg, *p* = 0.115), IOPcc (13.8 ± 13.6 mmHg, *p* = 0.772), and IOPg (12.9 ± 13.9 mmHg, *p* = 0.772). However, CRF was significantly lower in the uveitis group (9.7 ± 1.9 mmHg) than in controls (10.8 ± 1.5 mmHg; *p* = 0.009) [12].

Turan-Vural et al. (2012) [13] included 51 adult patients (mean age: 38.1 ± 14.5 years) with recurrent AU, all in inactive phase at the time of assessment. Etiologies included BD (37.3%), HLA-B27 AU (23.5%), sarcoidosis (17.6%), multiple sclerosis (11.8%), and idiopathic AU (9.8%). Compared to 34 healthy controls, patients showed significantly lower CH (8.57 ± 1.60 mmHg vs. 10.91 ± 1.41 mmHg; *p* = 0.001) and CRF (9.24 ± 1.68 mmHg vs. 11.56 ± 1.46 mmHg; *p* = 0.001). No significant differences were found in IOPcc (15.49 vs. 15.59 mmHg, *p* = 0.798) or IOPg (13.58 vs. 13.13 mmHg, *p* = 0.194) [13].

Sen et al. (2016) [16] analyzed 38 patients with Fuchs uveitis syndrome (mean age: 35.2 ± 12.9 years; all chronic cases). CH and CRF were significantly lower in affected eyes compared to both fellow non-affected eyes and healthy controls. CH was 9.5 ± 1.6 mmHg in affected eyes vs. 10.1 ± 1.7 mmHg in contralateral eyes (*p* = 0.005) and 10.5 ± 1.5 mmHg in controls (*p* = 0.005). CRF was 9.0 ± 1.9 mmHg in affected eyes vs. 9.9 ± 1.7 mmHg in contralateral eyes (*p* < 0.001) and 10.3 ± 1.5 mmHg in controls (*p* = 0.001). IOPg was significantly lower in affected eyes (13.1 ± 4.3 mmHg) compared to both controls (14.8 ± 2.5 mmHg, *p* = 0.025) and fellow eyes (14.6 ± 3.4 mmHg, *p* = 0.022). Regarding IOPcc, no significant differences were found in IOPcc between Fuchs-affected eyes (14.8 ± 4.1 mmHg), unaffected contralateral eyes (15.5 ± 3.4 mmHg, *p* = 0.231), and healthy controls (15.0 ± 2.7 mmHg, *p* = 0.766) [16].

Borrego-Sanz et al. (2023) included 77 adult patients with non-infectious uveitis (mean age: 52.68 ± 17.91 years), of which 22.1% were in the active phase and 77.9% were inactive. Etiologies included HLA-B27 (18.2%), idiopathic AU (16.9%), BD (9.1%), multifocal choroiditis, juvenile idiopathic arthritis, Vogt-Koyanagi-Harada disease, sarcoidosis, and Fuchs uveitis syndrome, among others. While CH, CRF and IOPg were not reported, IOPcc values were significantly lower in the uveitis group compared to healthy controls (13.57 mmHg vs. 15.28 mmHg; *p* < 0.002) [14].

Cankaya et al. (2014) studied 34 patients with ocular BD (mean age: 33.81 ± 9.36 years), of whom 47.1% were in the active phase. CH did not differ significantly compared to 20 healthy controls and 18 patients with inactive BD (8.51 vs. 8.47 mmHg, *p* > 0.05). CRF was higher in the active BD group (9.72) compared to the inactive (8.45) and control (8.43) groups, *p* < 0.05. IOPcc values were similar across all groups, but IOPg was significantly higher in patients with active BD (19.8 mmHg) compared to those with inactive BD (15.89 mmHg) and controls (15.59 mmHg), *p* < 0.05 [15].

Given the variability observed among individual studies, a meta-analysis was performed to derive pooled effect estimates of corneal biomechanical metrics in patients with ACI.

### Corneal biomechanics concerning activity and course of anterior chamber inflammation

Although it was not possible to conduct a separate statistical or meta-analytic evaluation based on the inflammatory activity or clinical course of ACI, individual studies reported corneal biomechanical alterations across various stages of the disease. Borrego-Sanz et al. compared eyes with active versus inactive ACI using Corvis ST^®^ and found significantly greater corneal deformability in the active group, as reflected by increased peak distance (PD: 5.16 ± 0.38 mm vs. 4.87 ± 0.37 mm; *p* = 0.007), deformation amplitude (DA: 1.24 ± 0.21 mm vs. 1.14 ± 0.10 mm; *p* = 0.007), applanation velocity 2 (V2: − 0.29 ± 0.04 m/s vs. − 0.26 ± 0.03 m/s; *p* = 0.018), and decreased applanation length 2 (L2: 1.69 ± 0.38 mm vs. 1.93 ± 0.34 mm; *p* = 0.015), suggesting increased corneal compliance during active inflammation [14]. In addition, Cem Cankaya et al. reported a significantly lower CRF in patients with inactive Behçet’s ocular disease (8.45 ± 1.98 mmHg) and healthy controls (8.43 ± 1.58 mmHg) compared with active Behçet’s ocular disease (9.72 ± 2.11 mmHg), *p* < 0.05) [15], suggesting a relative decrease in structural rigidity during inactive periods, possibly due to subclinical or cumulative remodeling.

Concerning the course, Sen et al. showed that corneal biomechanical alterations are present in chronic ACI. For example, in patients with unilateral chronic Fuchs’ uveitis, CH and CRF in affected eyes were significantly lower than in fellow unaffected eyes (CH: 9.5 ± 1.6 mmHg vs. 10.1 ± 1.7 mmHg; *p* = 0.005; CRF: 9.0 ± 1.9 mmHg vs. 9.9 ± 1.7 mmHg; *p* < 0.001) and healthy controls (CH: 10.5 ± 1.5 mmHg; CRF: 10.3 ± 1.5 mmHg; *p* = 0.005 and 0.001, respectively)​ [16]. Similarly, Turan-Vural et al. evaluated 51 patients with recurrent but inactive anterior uveitis and found significantly lower CH (8.57 ± 1.60 mmHg vs. 10.91 ± 1.41 mmHg; *p* < 0.001) and CRF (9.24 ± 1.68 mmHg vs. 11.56 ± 1.46 mmHg; *p* < 0.001) compared to healthy controls [13], suggesting that chronic or recurrent inflammation may induce structural changes independent of clinical activity.

### Meta-analysis

Meta-analyses were conducted for four parameters: CH, CRF, IOPg, and IOPcc between ACI patients and healthy controls. The initial analyses revealed high heterogeneity across studies for CH (I² = 83%), CRF (I² = 88%), and IOPg (I² = 89%), while IOPcc demonstrated no heterogeneity (I² = 0%) (Supplementary Material 3).

A sensitivity analysis was conducted to explore the source of heterogeneity. One study (Cankaya et al.) was initially flagged as a potential outlier based on its markedly divergent result in the forest plot for CRF. This was further confirmed through a leave-one-out analysis, which showed that excluding this study reduced heterogeneity in the CRF model from 88 to 69% and significantly altered the pooled effect estimate, indicating that the study had a disproportionate influence on both the magnitude of the pooled effect and the overall heterogeneity [21, 22]. Based on this combined evidence, the study was excluded from the meta-analysis of CRF to improve robustness and interpretability.

**Fig. 3:**
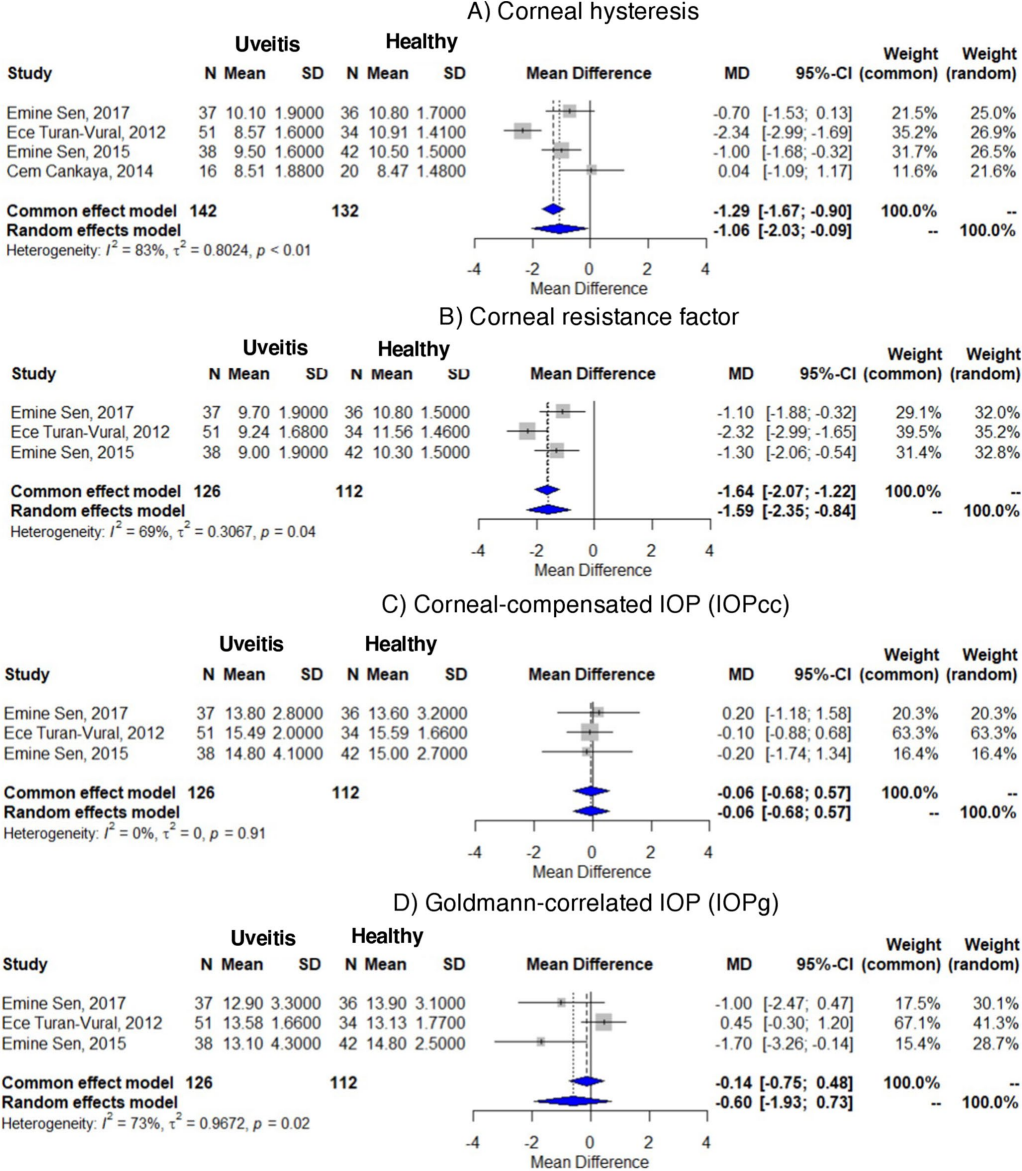
Differences in corneal biomechanical properties between patients with anterior chamber inflammation and healthy controls. N: Number of patients, SD: Standard deviation, MD: Mean difference, CI: Confidence interval

The random effects model revealed that CH was significantly reduced by −1.06 mmHg in patients with ACI compared to control subjects [95% CI: −2.03, −0.09] (I² = 83%). Similarly, CRF was − 1.59 mmHg lower in ACI patients than in healthy controls [95% CI: −2.35, −0.84] (I² = 69%), suggesting significant alterations in biomechanical properties in ACI patients compared to healthy controls. No significant differences were observed for IOPcc [−0.06 mmHg; 95% CI: −0.68, 0.57] (I² = 73%) or IOPg [−0.60 mmHg; 95% CI: −1.93, 0.73] (I² = 73%) (Fig. 3).

**Fig. 4:**
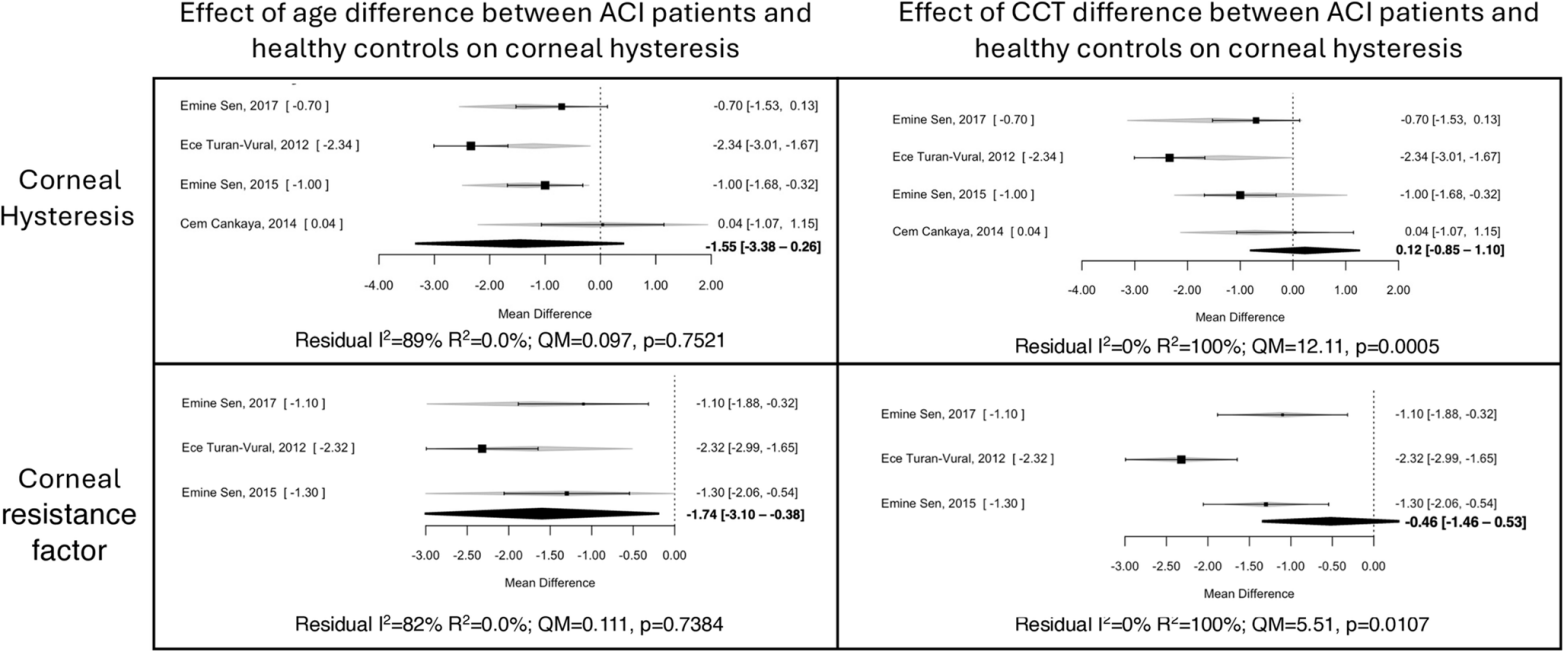
Meta-regression: Effect of age and CCT differences between ACI patients and controls as moderators of CH and CRF outcomes. Residual I²: Heterogeneity in the model that remains unexplained by the moderator. R²: Proportion of heterogeneity explained by the moderator. QM (Moderator test): Test assessing whether the heterogeneity explained by the moderator is significant (*p* < 0.05)

Due to age and CCT affecting biomechanical properties, a second metanalysis was performed for CH and CRF, including them as moderators. Regarding the CH, the significance was lost for both variables: age [−1.55; 95% CI: −3.38, 0.26] and CCT [0.12; 95% CI: −0.85, 1.10]. However, there were notable differences in the heterogeneity explained by the model with each moderator. For age, the moderator did not account for any heterogeneity (QM = 0.097, *p* = 0.7521; R²=0%), and the residual model’s heterogeneity remained high (I²=89%), indicating that age did not influence the results. In contrast, CCT as a moderator explained all the heterogeneity (R²=100%; QM = 12.11, *p* = 0.0005), reducing the I² to 0% (Fig. 4), suggesting that differences in CCT may be associated with differences in biomechanical outcomes in patients with ACI.

For the CRF results with age and CCT as moderators, the lower CRF remained significant when age was the moderator [−1.74; 95% CI: −3.10, −0.38] but not when moderated by CCT [−0.46; 95% CI: −1.46, 0.53]. This suggests that a lower CRF may provide a more reliable measure of corneal weakening in patients with ACI, consistently across all ages. Similar to the findings for CH, the age moderator did not account for any heterogeneity (QM = 0.111, *p* = 0.7384; R²=0%), whereas CCT explained all the heterogeneity (QM = 5.51, *p* = 0.0107; R²=100%) (Fig. 4). This supports the fact that CCT markedly influences both CH and CRF measures.

## Discussion

The present study identified a significant reduction in CH and CRF in patients with ACI compared to healthy controls. Among the included studies, all reported reduced CH and CRF values in ACI patients, with one exception: only the study by Cem Cankaya et al. documented higher CRF values in the active uveitis group compared to both inactive patients and healthy controls. We hypothesize that this finding may be influenced by the positive correlation between CCT and CRF, as further discussed below. However, it is worth noting that the CRF values reported for the healthy control group in Cankaya’s study were unusually low (8.43 ± 1.58 mmHg) when compared to those reported in other populations. For instance, Hurmeric et al. found a mean CRF of 10.1 ± 1.8 mmHg in healthy eyes [23], Narayanaswamy et al. reported a mean CRF of 10.1 ± 1.6 mmHg in a Chinese adult population [24], and Chua et al. documented a mean CRF of 10.3 ± 1.7 mmHg in both Chinese and Indian adults [25]. These discrepancies may reflect differences in measurement conditions, population characteristics, or device calibration, and could have contributed to the apparent elevation of CRF in the active uveitis group within that specific study.

Although assessing the biomechanical properties of the cornea is not part of the routine evaluation for uveitis patients, these findings highlight areas that warrant further research, such as their role in preoperative assessments for refractive surgery and the potential of these properties as indirect measures of inflammation.

### Impact of biomechanical properties in preoperative assessment for refractive surgery in patients with anterior chamber inflammation

Regarding the preoperative assessment for refractive surgery, although the overall safety of these procedures is relatively high, between 0.04% and 0.6% of patients develop post-surgical ectasia [26]. This subset of patients is believed to have biomechanically abnormal corneas before surgery, often in the form of subclinical keratoconus [27, 28]. For example, in 26 eyes with highly asymmetrical corneal ectasia, CH was significantly altered (*p* = 0.04). Although corneas are thoroughly screened before refractive surgery using topographic, tomographic, and pachymetric evaluations and, more recently, through genetic testing [29–31], none of the current screening tools can reliably detect biomechanical instability. This study identified alterations in corneal biomechanical properties in patients with ACI. Notably, some of the included studies focused exclusively on inactive ACI. Refractive surgeons should be aware of the potential risk of biomechanical weakening, even in patients with a history of ACI. The use of biomechanical assessment devices during the preoperative evaluation of patients with inactive ACI could potentially enhance screening protocols by identifying subclinical alterations that may not be detected through standard examinations.

### Impact of age and CCT on biomechanical properties in patients with anterior chamber inflammation

It is well established that corneal biomechanical properties vary with age, with both CH and CRF decreasing as age increases [32]. In the analysis considering the age difference between the group with ACI and the healthy group, CRF remained significantly lower in patients with ACI, suggesting that CRF may be a more specific indicator than CH, regardless of age. Although the significance of CH was lost in the analysis [−1.55; 95% CI: −3.38, 0.26], the impact of age on the model was not significant either (R²=0.0%; QM = 0.097, *p* = 0.7521), leaving a high residual heterogeneity and limiting the conclusions that can be drawn. Consequently, significant differences in CRF, but not CH, have been observed in pediatric ACI compared to healthy controls [12]. In contrast, in adults with ACI, a significant reduction in both (CH and CRF) has been observed, highlighting the need for further studies [13, 14].

Interestingly, when the difference in CCT between groups was used as a moderator, it explained 100% of the heterogeneity for both CH and CRF, highlighting that differences in CCT may be associated with differences in biomechanical outcomes. A positive correlation between CCT, CH, and CRF has been reported in healthy individuals and uveitis patients, indicating that thicker corneas generally exhibit greater biomechanical stiffness [12].

In the systematic review by Mejía-Salgado et al. [10], CCT was significantly increased in patients with ACI compared to healthy controls, particularly during active inflammation and acute episodes. It was proposed that, similar to retinal nerve fiber layer thickness during acute episodes of posterior uveitis [33, 34], CCT may serve as a potential indicator of inflammatory activity in the anterior chamber [10].

We hypothesize that the increase in CCT may lead to a corresponding elevation in CH and CRF in acute episodes of active inflammation. For instance, Cem Cankaya et al. reported that patients with active ocular Behçet’s disease had significantly higher CCT (592.50 ± 39.95 μm) and CRF (9.72 ± 2.11 mmHg) values compared to those with inactive disease (528.35 ± 19.00 μm and 8.45 ± 1.98 mmHg, respectively) and healthy controls (526.30 ± 18.21 μm and 8.43 ± 1.58 mmHg, respectively) *p* < 0.05 [15]. However, this increase in CCT, being the result of inflammation-induced stromal swelling rather than an increase in stromal layering, does not lead to greater rigidity but rather to increased corneal distensibility. Supporting this, Borrego-Sanz et al. found that eyes with ACI demonstrated increased peak distance, deformation amplitude, and applanation velocity 2, along with decreased applanation length 2, compared to inactive eyes—findings that are indicative of corneal biomechanical weakening [14].

Conversely, during remission, when endothelial cell damage and dysfunction have been documented [10], the subsequent influx of aqueous humor may lead to a subtle stromal edema and reduced viscoelasticity, resulting in lower CH and CRF values. This pattern resembles that observed in postoperative corneal edema, where CH has been shown to decrease significantly [35]. In line with this, the study by Sen et al. [16], which included only patients with inactive Fuchs uveitis syndrome, also reported reduced CH and CRF, suggesting that subclinical or residual biomechanical alterations may persist even in the absence of clinically evident inflammation.

#### Assessing IOP in anterior chamber inflammation: are non-contact parameters superior?

The previously reported high CCT values during acute active episodes have raised concerns about potential differences in IOP measurements using contact versus non-contact tonometers in patients with ACI [10]. In the current study, no significant differences were observed between IOPcc and IOPg in the ACI group; however, the result was borderline significant (*p* = 0.051). Given the small sample size and the inability to differentiate between active and inactive or acute versus recurrent chronic cases, where variations in CCT are more pronounced, further studies are needed to determine whether non-contact tonometers may perform better than contact tonometers during active episodes of ACI.

This study has limitations. Notably, all the studies included in the meta-analysis were observational, which inherently increases the risk of selection, confounding, or interpretation bias. However, all the studies utilized the same equipment (ORA) and had similar controls (healthy groups), which enhanced the reliability of the results. Additionally, we conducted a sensitivity analysis, excluding studies with outlier values. This approach reduced heterogeneity in the CRF model from 88 to 69%. Nevertheless, heterogeneity remained high for other parameters, such as CH (I² = 83%) and IOPg (I² = 73%), highlighting the need for future studies in more homogeneous populations to allow for more definitive conclusions.

Furthermore, there is limited literature on biomechanical measurements in ACI, as evidenced by the small number of included studies and their high heterogeneity. This systematic review summarizes the available literature; however, further research is needed to improve the understanding of biomechanical changes concerning uveitis characteristics, such as activity (active vs. remission), course (acute, recurrent, chronic), and involvement of other anatomical locations (without ACI involvement).

In conclusion, patients with ACI exhibit a reduction in corneal biomechanical properties. The decrease in CRF appears consistent across all age groups in ACI patients, whereas the reduction in CH is more pronounced in adult populations. No significant variations were observed in IOPg and IOPcc among ACI patients. These findings pave the way for further research, including the impact of biomechanical alterations on vision in ACI patients, their role in preoperative assessments, and the monitoring of patients with ACI.

## Supplementary Information

Below is the link to the electronic supplementary material.

Supplementary Material 1: PRISMA checklist

Supplementary Material 2: Search Strategy

Supplementary Material 3: Sensitivity analysis

High Resolution Image

## Data Availability

This study is a secondary source analysis that relies on compiling previously published data. Information. Results can be obtained by conducting the search strategy in Supplementary Material 2, sensitive analysis in Supplementary Material 3, and meta-analyses.
