# Supplementary material for: Corneal biomechanical changes in patients with anterior chamber inflammation: a systematic review and meta-analysis
Source: Graefes Arch Clin Exp Ophthalmol. 2025 Sep 11;263(12):3447–60. doi: 10.1007/s00417-025-06881-y (PMC12886276; doi:10.1007/s00417-025-06881-y)
Supplement: Supplementary file 2 — Supplementary Material 2: Search Strategy [file 417_2025_6881_MOESM2_ESM.docx]

**Search Strategy**

SEARCH STRATEGY PUBMED-DATE MAY 02^TH^, 2024 14:30 PM

|  | **LINE** | **ARTICLES** |
| --- | --- | --- |
| **POPULATION** | (uveitis[MeSH Terms]) OR (uveitis[Title/Abstract])) OR (uveitis, anterior[Title/Abstract])) OR (Iridocyclitis[MeSH Terms])) OR (Iridocyclitis[Title/Abstract])) OR (Iritis[MeSH Terms])) OR (Iritis[Title/Abstract]) | 44,423 |
| **EXPOUSURE** | (Corneal Biomechanics[All Fields] OR Corneal Biomechanical Properties[All Fields] OR "Ocular Response Analyzer"[All Fields] OR Corneal Hysteresis[All Fields] OR Corneal Resistance Factor[All Fields] OR "Corneal Compensated IOP"[All Fields] OR "Corvis ST"[All Fields] OR "Shear Modulus"[All Fields] OR "Elastic Modulus"[All Fields] OR "Young’s Modulus"[All Fields] OR "Viscoelastic Properties"[All Fields] OR Pachymetry[All Fields] OR "Deformation Response"[All Fields] OR Bioengineering[All Fields] OR "Biophysical Properties"[All Fields]) | 533,314 |
| **SEARCH STRATEGY** | ((((((uveitis[MeSH Terms]) OR (uveitis[Title/Abstract])) OR (uveitis, anterior[Title/Abstract])) OR (Iridocyclitis[MeSH Terms])) OR (Iridocyclitis[Title/Abstract])) OR (Iritis[MeSH Terms])) OR (Iritis[Title/Abstract])) AND (Corneal Biomechanics[All Fields] OR Corneal Biomechanical Properties[All Fields] OR "Ocular Response Analyzer"[All Fields] OR Corneal Hysteresis[All Fields] OR Corneal Resistance Factor[All Fields] OR "Corneal Compensated IOP"[All Fields] OR "Corvis ST"[All Fields] OR "Shear Modulus"[All Fields] OR "Elastic Modulus"[All Fields] OR "Young’s Modulus"[All Fields] OR "Viscoelastic Properties"[All Fields] OR Pachymetry[All Fields] OR "Deformation Response"[All Fields] OR Bioengineering[All Fields] OR "Biophysical Properties"[All Fields]) | 162 |

SEARCH STRATEGY EMBASE-DATE MAY 02^TH^, 2024 14:35 PM

|  | **LINE** | **ARTICLES** |
| --- | --- | --- |
| **POPULATION** | ('uveitis'/exp OR uveitis OR 'uveitis, anterior' OR 'iridocyclitis'/exp OR iridocyclitis OR 'iritis'/exp OR iritis) | 79,022 |
| **EXPOUSURE** | ('corneal biomechanics' OR 'corneal biomechanical properties' OR 'ocular response analyzer' OR 'corneal hysteresis' OR 'corneal resistance factor' OR 'corneal compensated iop' OR 'corvis st' OR 'shear modulus' OR 'elastic modulus' OR 'young modulus' OR 'viscoelastic properties' OR pachymetry OR 'deformation response' OR bioengineering OR 'biophysical properties') | 275,694 |
| **SEARCH STRATEGY** | ('uveitis'/exp OR uveitis OR 'uveitis, anterior' OR 'iridocyclitis'/exp OR iridocyclitis OR 'iritis'/exp OR iritis) AND ('corneal biomechanics' OR 'corneal biomechanical properties' OR 'ocular response analyzer' OR 'corneal hysteresis' OR 'corneal resistance factor' OR 'corneal compensated iop' OR 'corvis st' OR 'shear modulus' OR 'elastic modulus' OR 'young modulus' OR 'viscoelastic properties' OR pachymetry OR 'deformation response' OR bioengineering OR 'biophysical properties') | 178 |

SEARCH STRATEGY VHL-DATE MAY 02^TH^, 2024 14:38 PM

|  | **LINE** | **ARTICLES** |
| --- | --- | --- |
| **POPULATION** | ('uveitis'/exp OR uveitis OR 'uveitis, anterior' OR 'iridocyclitis'/exp OR iridocyclitis OR 'iritis'/exp OR iritis) | 31,725 |
| **EXPOUSURE** | ("corneal biomechanics" OR "corneal biomechanical properties" OR "ocular response analyzer" OR "corneal hysteresis" OR "corneal resistance factor" OR "corneal compensated iop" OR "corvis st" OR "shear modulus" OR "elastic modulus" OR "young modulus" OR "viscoelastic properties" OR "pachymetry" OR "deformation response" OR "bioengineering" OR "biophysical properties") | 64,702 |
| **SEARCH STRATEGY** | (mh:"Uveitis" OR "uveitis" OR "uveitis, anterior" OR mh:"Iridocyclitis" OR "iridocyclitis" OR mh:"Iritis" OR "iritis") AND ("corneal biomechanics" OR "corneal biomechanical properties" OR "ocular response analyzer" OR "corneal hysteresis" OR "corneal resistance factor" OR "corneal compensated iop" OR "corvis st" OR "shear modulus" OR "elastic modulus" OR "young modulus" OR "viscoelastic properties" OR "pachymetry" OR "deformation response" OR "bioengineering" OR "biophysical properties") | 32 |

SEARCH STRATEGY MEDXRIVDATE MAY 02^TH^, 2024 14:45 PM

|  | **LINE** | **ARTICLES** |
| --- | --- | --- |
| **POPULATION** | ("uveitis" OR "iridocyclitis" OR "iritis") | 165 |
| **EXPOUSURE** | ("corneal biomechanics” OR "ocular response analyzer") | 47,614 |
| **SEARCH STRATEGY** | ("uveitis" OR "iridocyclitis" OR "iritis") AND ("corneal biomechanics" OR "ocular response analyzer") | 0 |
