# Supplementary figures and images for: Corneal biomechanical changes in patients with anterior chamber inflammation: a systematic review and meta-analysis
Source: Graefes Arch Clin Exp Ophthalmol. 2025 Sep 11;263(12):3447–60. doi: 10.1007/s00417-025-06881-y (PMC12886276; doi:10.1007/s00417-025-06881-y)

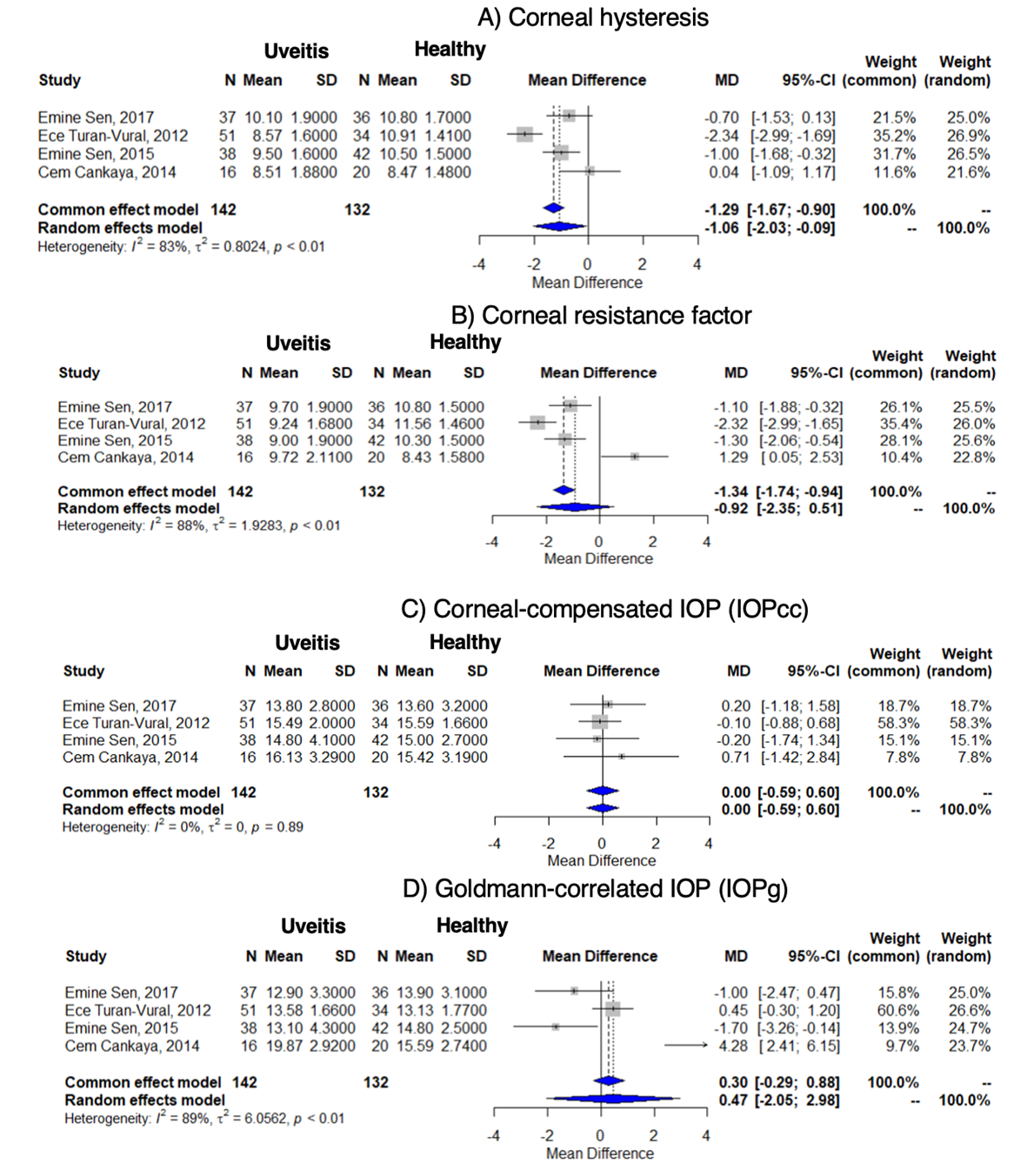

Supplement: Supplementary file 3 — Sensitivity analysis [file 417_2025_6881_Fig5_ESM.png]

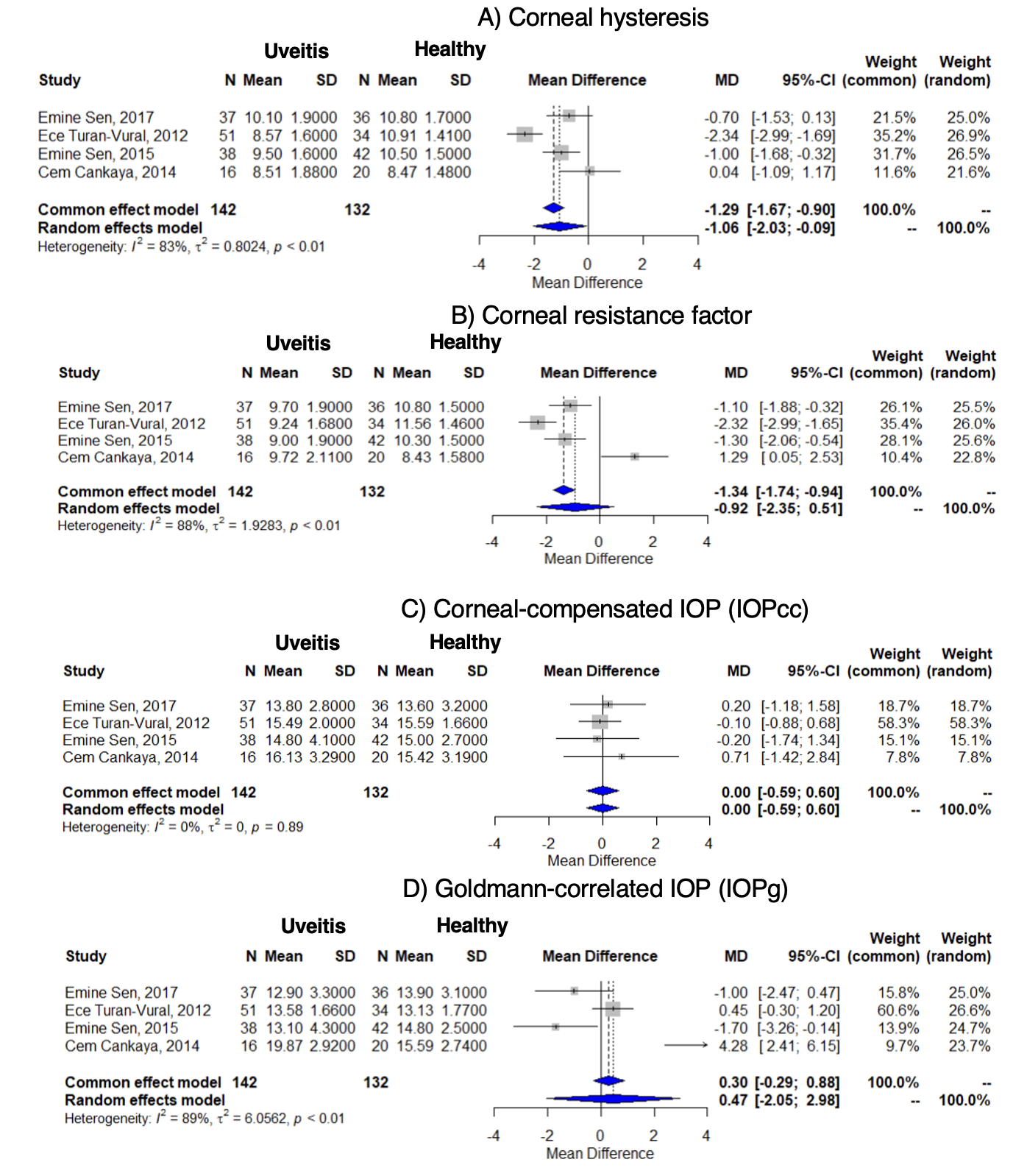

Supplement: Supplementary file 4 — High Resolution Image [file 417_2025_6881_MOESM3_ESM.tif]
